# Ets-2 Acts As a Transcriptional Repressor of the Human Immunodeficiency Virus Type 1 through Binding to a Repressor–Activator Target Sequence of 5′-LTR

**DOI:** 10.3389/fimmu.2017.01924

**Published:** 2018-01-04

**Authors:** Ioannis Panagoulias, Fotios Karagiannis, Ioanna Aggeletopoulou, Tassos Georgakopoulos, Christos P. Argyropoulos, Karolina Akinosoglou, Charalambos Gogos, Athanasios Skoutelis, Athanasia Mouzaki

**Affiliations:** ^1^Division of Hematology, Department of Internal Medicine, Medical School, University of Patras, Patras, Greece; ^2^Division of Nephrology, Department of Internal Medicine, Medical School, University of New Mexico, Albuquerque, NM, United States; ^3^Infectious Diseases Unit, Department of Internal Medicine, University Hospital of Patras, Patras, Greece; ^4^Department of Internal Medicine and Infectious Diseases Unit, Evangelismos General Hospital, Athens, Greece

**Keywords:** HIV-1, Ets-2, repressor–activator target sequence, transcription factors, repressor, T helper cells, naive T cells, viral latency

## Abstract

HIV-1 is transcriptionally active in activated T helper (Th)-cells and inactive in naive or resting memory Th-cells. Ets-2 is a preinduction transcriptional repressor of the IL-2 gene in naive Th-cells and a candidate transcriptional repressor of HIV-1 in the same cells, because the −279 to −250 upstream region of HIV-1-LTR [repressor–activator target sequence (RATS)], that participates in HIV-1-LTR transcriptional silencing, encompasses the AAGGAG Ets-2 binding site. In this proof of concept study, we investigated whether Ets-2 represses the expression of HIV-1. To assess whether Ets-2 can repress HIV-1 transcriptional activation acting through RATS, we transfected Jurkat cells with an Ets-2 overexpression plasmid (pCDNA3-ets-2) or Ets-2 silencing plasmids (ets-2-shRNA) and, as target genes, plasmids carrying the whole HIV-1-LTR sequence (HIV-1-LTR-CAT) or two copies of the RATS sequence (2× RATS-CAT) or a point mutation in the Ets-2 binding site (2× mutantRATS-CAT) or CMV-CAT (control). Ets-2 overexpression resulted in a significant reduction of HIV-1-LTR-CAT and 2× RATS-CAT activities in stimulated cells, but not of the 2× mutantRATS-CAT or CMV-CAT. Ets-2 silencing led to increased activities of HIV-1-LTR-CAT and 2× RATS-CAT in unstimulated cells, but had no effect on the activities of 2× mutantRATS-CAT and CMV-CAT. To assess Ets-2 binding to HIV-1-LTR–RATS in naive Th-cells, we isolated naive Th-cell nuclear proteins and passed them through an Ets-2 antibody column; electrophoretic mobility shift assays were performed using an RATS probe mixed with consecutive protein eluates. Ets-2 bound to the HIV-1-LTR–RATS in a dose-dependent manner. To assess Ets-2 binding to RATS *in vivo*, Jurkat cells were transfected with 2× RATS-CAT and stained for the Ets-2 protein and the RATS sequence by combining immunofluorescence and fluorescence *in situ* hybridization techniques. In unstimulated cells, Ets-2 bound to RATS, whereas no binding was observed in stimulated cells. To test for RATS specificity, the same experiments were performed with 2× mutantRATS-CAT, and no binding of Ets-2 was observed. The results were corroborated by chromatin immunoprecipitation assays performed with the same cells. Our results show that Ets-2 is a transcriptional repressor of HIV-1. Repression of HIV-LTR-RATS mediated by Ets-2 may account for the low-level transcription and replication of HIV-1 in naive Th-cells, and contribute to the viral latency and maintenance of viral reservoirs in patients, despite long-term therapy.

## Introduction

HIV-1 displays tropism for T helper (Th) cells, and the progression of the disease is directly related to Th-cell death. Despite initial hopes that HAART delivery might be able to affect a cure, further studies revealed that it did not completely eliminate HIV from the plasma and persistent viremia could be detected in the individuals even after 8 years of HAART therapy (1–4). Viral relapse occurs due to the maintenance of latent viral reservoirs that are capable of resuming the infection (3). So far, three types of cellular reservoirs have been identified: naive and resting memory Th cells (4–6), monocytes and macrophages (7–9), and myeloid and follicular dendritic cells (10, 11). Regarding Th cells, although the HIV-1 virus infects naive and resting memory Th cells, naive Th cells expressing the CD45RA surface marker are not permissive to viral expression and replication (6, 12).

HIV-1 transcription or latency depends on an elaborate interplay between the chromatin integration site of the virus, viral, and host transcriptional factors and chromatin modifications (13–15). Numerous host transcriptional factors including NFAT, NF-κB p50/p65, AP-1, and SP-1 (15–17) contribute to viral transcriptional activation through direct binding to the virus long terminal repeat (5′-LTR) that acts as a transcriptional promoter (15, 17). Other host factors, including YY1, NF-κB p50/p50, CBF-1, STAT5, MBP-1, Foxp3, and ZBRK, contribute to the transcriptional repression of the virus indirectly, by disrupting molecular pathways that result in viral activation (16, 18–24).

The 5′-LTR region of HIV-1 encompasses a negative regulatory element (NRE) spanning −340 to −185 nucleotides upstream the transcription initiation site (25). NRE encompasses a repressor–activator target sequence (RATS) element (−279 to −250) (26, 27), the sequence of which shares significant homology to the antigen receptor response element 2 (ARRE-2) of the IL-2 gene promoter (27, 28). Deletion of the NRE region from the LTR sequence in a Jurkat-tat cell line led to induction of viral transcription (25); conversely, the insertion of a copy of the NRE sequence under the control of a heterologous promoter led to reduction of viral transcription (28). Our earlier transactivation studies in the *Xenopus laevis* oocyte system showed that nuclear protein extracts isolated from peripheral blood naive Th cells exerted a strong repression activity on the expression of CAT reporter genes under the control of the HIV-1-LTR or the RATS element (27). This repression activity was counteracted by the addition of nuclear protein extracts isolated from activated Th cells, leading to the derepression of the HIV-1-LTR-CAT and RATS-CAT genes. In addition, we showed that the repression activity was not observed when nuclear protein extracts isolated from resting memory Th cells were used in the experiments (27). Based on these observations, we hypothesized that a transcriptional repressor is present in naive Th cells, which binds to the RATS sequence of HIV-1-LTR and represses HIV-1 expression. An *in silico* analysis we performed to mine microarray data to identify transcription factors expressed in naive Th cells but not in activated Th cells, revealed that the transcription factor Ets-2 was the strongest candidate for being the transcriptional repressor (29).

Ets-2 belongs to the Ets (E26 transformation specific) family of transcription factors that have a characteristic winged helix-turn-helix DNA-binding domain and bind to a core GGAA/T consensus sequence (30–32). Ets factors are involved in the transcriptional regulation of several genes and play an important role in various cellular functions (mitosis, growth, development, differentiation, and apoptosis) and the regulation of immunity (33, 34).

Ets-2 is expressed during the early stages of human T lymphocyte development (35) and plays a protective role in the proliferation, maturation, and survival of mouse thymocytes (36). Ets-2 has the ability to activate or repress the transcription of specific target genes. In the breast cancer cell line MCF-7, overexpression of exogenous Ets-2 leads to the repression of transcription of the endogenous BRCA1 gene through direct binding to its promoter (37). Ets-2 has also been found to have a tumor suppressor role in a mouse model of Down syndrome in which enhanced Ets-2 activity induced significant inhibition of intestinal tumors (38).

Recently, we showed that IL-2 expression is blocked in human naive, but not activated or memory Th cells, by the transcription factor Ets-2 that binds to ARRE-2 of the proximal IL-2 promoter (39). In particular, we demonstrated that Ets-2 acts as an independent preinduction repressor exclusively in naive Th cells and does not interact physically with the transcription factor NFAT that binds to the ARRE-2 in activated Th cells. In naive Th cells, Ets-2 mRNA expression, Ets-2 protein levels, and Ets-2 binding to ARRE-2 decrease upon cell activation, followed by the concomitant expression of IL-2. Ets-2 silences directly constitutive or induced IL-2 expression through the ARRE-2; conversely, Ets-2 silencing allows for constitutive IL-2 expression in unstimulated cells. Ets-2 binding to ARRE-2 in chromatin is stronger in naive compared with activated or memory Th cells; in the latter, Ets-2 participates in a change of the IL-2 promoter architecture, possibly to facilitate a quick response when the cells re-encounter antigen. In addition, we showed that cyclosporine A stabilizes Ets-2 mRNA and protein when the cells are activated (revealing a new function of cyclosporine A) (39).

In this proof of concept study, we show that Ets-2 represses HIV-1 transcription through binding to the RATS element of the LTR. Thus, we provide solid evidence for a hitherto unknown significant parameter that may contribute to the viral latency and maintenance of viral reservoirs in naive Th cells of patients, despite long-term therapy.

## Materials and Methods

### Cells, Phenotyping, and Cultures

#### Primary Cells

Peripheral blood samples (10–20 ml) from 20 healthy young adults (13 F/7 M, age range 22–35 years) were collected in heparinized tubes. Peripheral blood mononuclear cells (PBMCs) were isolated by centrifugation of whole blood over a Ficoll-Paque gradient (Biochrom) and washed 4× with ice-cold RPMI1640 culture medium (Gibco). CD19+ B cells, CD14+ monocytes, CD3+ T cells, CD4+ CD25− Th cells, CD4+ CD25+ Th cells, CD8+ T cytotoxic cells, CD4+ CD45RA+ CD25− naive Th cells, and CD4CD45RO+ CD25− memory Th cells were isolated by cell sorting using a BD FACS Aria II flow cytometer (BD Biosciences). The sorting strategy is shown in Figure S1 Supplementary Material. The isolated cell populations were phenotyped and used when their purity reached >95%. The antibodies used for cell sorting and phenotyping were the mouse antihuman monoclonal antibodies (mAbs) CD3-APC-H7 (clone SK7), CD19-APC (clone HIB19), CD14-FITC (clone M5E2), CD4-APC (clone RPA-T4), CD8-FITC (clone HIT8a), CD25-PE (clone M-A251), CD45RA-APC-H7 (clone 5H9), and CD45RO-PE-Cy7 (clone UCHL1) (BD Biosciences), CD25-PC5 (clone B1.49.9) (Beckman Coulter). Fluorescence minus one controls were used to identify any background spread of fluorochromes and establish gating boundaries. The data were analyzed using the BD FACS DIVA software v.8.

#### Cell Lines

The cell lines used were Jurkat (T-cell acute lymphoblastic leukemia), U937 (histiocytic lymphoma) (American Type Culture Collection), RPMI8866 (B lymphoid cell line), and Raji (Burkitt’s lymphoma) (European Collection of Cell Cultures).

#### Cell Culture

Primary cells and cell lines were cultured in RPMI1640 medium supplemented with 10% fetal bovine serum (FBS), penicillin (100 U/ml), and 50 µM 2-mercaptoethanol (CM), at a concentration of 10^6^ cells/ml, in a 37°C humidified chamber with 5% CO_2_. When required, the cells were stimulated with the mitogens ionomycin (2 μM) and phorbol myristate acetate (20 ng/ml) (P/I) for the times indicated.

### Quantitative Real-time PCR

Total RNA was isolated by the standard Trizol method according to the manufacturer’s instructions (Gibco). The RNA yield and purity were determined by measuring absorbance at 260/280 nm on a Quawell micro volume spectrophotometer Q3000 (Quawell Technology). Total RNA (250 ng per experimental point) was reverse transcribed with M-MLV reverse transcriptase (200 U/μl) (Sigma-Aldrich) in 10× M-MLV Reverse Transcriptase Buffer, 40 U/μl RNase inhibitor, 1 mM each of the dNTPs, and 2.5 µM random hexanucleotide primers (Sigma-Aldrich). Quantitative real-time PCR mRNA analysis was performed on an Mx3000P_TM_ Quantitative PCR System thermal cycler (Stratagene), using the SYBR-green fluorescence quantification technology (KAPA SYBR FAST qPCR Kit, Kapa Biosystems). PCR conditions were 95°C for 15 min followed by 40 cycles of 95°C for 30 s to denature the cDNA, 58°C for 30 s for annealing, and 72°C for 30 s for extension. The results were analyzed using the MxPro_TM_ software (Stratagene). Expression of β2-m gene served as the normalizer. The primers for ets-2 were as follows: 5′-CTCGTGTGTCTCAACCATCTT-3′ and 5′-CGCTCTGTGCCTCAGAATAG 3′, yielding a 112 bp PCR product and for β2-m 5′-TCGCGCTACTCTCTCTTTCT-3′ and 5′-TTTCCATTCTCTGCTGGATGAC-3′, yielding an 88 bp PCR product. All measurements were done in triplicate.

### Western Immunoblotting

Whole cell extracts (10^6^ cells per experimental point) were prepared using RIPA buffer (50 mM Tris–HCl pH 7.4, 150 mM NaCl, 1% Triton X-100, 1% sodium deoxycholate, 0.1% SDS, and 1 mM EDTA) supplemented with protease inhibitors (Sigma-Aldrich). Protein concentration was determined using the Bradford Assay (Sigma-Aldrich). The protein extracts (10 µg per experimental point) were separated by SDS-PAGE and electrophoretically transferred to PVDF membranes. The membranes were incubated with a rabbit polyclonal Ab to human Ets-2 (Santa Cruz Biotechnology). A mouse mAb to human β-tubulin (Upstate Biotechnology) served as a loading control. The membranes were incubated with HRP-conjugated goat anti-rabbit IgG (Upstate Biotechnology) and goat anti-mouse IgG (Upstate Biotechnology) Abs, respectively. Protein levels were visualized with the ECL LumiGLO detection kit (Upstate Biotechnology).

### Immunofluorescence

Naive and memory Th cells and Jurkat cells were attached on poly-l-lysine coated slides (10^5^ cells per slide), rinsed with PBS and fixed with 4% PFA for 10 min at RT. Fixed cells were permeabilized with 0.3% Triton X-100 in PBS for 4 min and incubated in blocking buffer (PBS with 10% FBS and 3% BSA) for 1 h at RT. The cells were stained with mouse anti-Ets-2 mAb (Santa Cruz, sc-373754) for 2 h at RT, washed two times with 0.05% Tween-20 in PBS (5 min per wash), and incubated with Alexa Fluor 488-conjugated goat anti-mouse Ab (Thermo Fisher, A-11001) for 1 h at RT. Cells were then counterstained with 4,6-diamidino-2-phenylindole (DAPI) (1 µg/ml) for 10 min, mounted in MOWIOL, and stored at 4°C in the dark. Images were recorded on a Leica TCSSP5 confocal microscope. Digital images were processed using FIJI software.

### Ets-2 Purification and Electrophoretic Mobility Shift Assays (EMSAs)

Ets-2 protein was isolated from 1 mg of nuclear protein extracts, derived from sorted CD4+ CD25− Th cells from all PBMC samples. The extracts were diluted 1:5 in KIB buffer (20 mM HEPES pH 7.6, 100 mM KCl, 0.02 mM EDTA, 0.5 mM 1,4-dithio-dl-threitol) and passed through a protein A/G agarose column. The eluate was mixed for 1 h with 20 µg Ets-2 Ab (Santa Cruz Biotechnology) and then passed through a protein A/G agarose column. Bound Ets-2 was eluted with ImmunoPure Gentle Ag/Ab Elution Buffer (Thermo Fisher Scientific) and collected in 1 ml aliquots. For EMSA, the proteins were precipitated by 10% w/v PEG8000, and the pellets were redissolved in KIB buffer; EMSAs were performed as described (39). The oligonucleotide probe used was the RATS sequence AGGCCAATGAAGGAGAGAACAACAGCTTGT (27).

### DNA Plasmids

An ets-2 expression plasmid, pcDNA3-ets-2, was generated as described (29, 39). The target genes used were as follows: pUC-BENN-CAT (40) (a gift from NIH), which contains the full-length LTR from HIV-1 (spanning nucleotides −450/+531) controlling a CAT-coding sequence followed by an SV40 polyadenylation signal, 2× RATS-CAT containing two copies of the RATS region of HIV-1-LTR (−279/−250) in front of a TK promoter, 2× mutantRATS-CAT containing two mutated copies of the RATS sequence [point mutation at the Ets-2 binding site (G/T)] of HIV-1-LTR in front of a TK promoter, and CMV-CAT as a control (27, 39).

For the ets-2 mRNA knockdown experiments five ets-2 shRNA plasmids that target five different sites of ets-2 mRNA were used (MISSION^®^ shRNA Plasmid DNA, Sigma-Aldrich).

### Cell Transfections

Jurkat, RPMI8866, Raji, and U937 cells were transfected with the pUC-BENN-CAT and CMV-CAT plasmids using Lipofectamine LTX DNA Transfection Reagents (Invitrogen) according to the manufacturer’s instructions. Jurkat cells were also co-transfected with stable amounts of the reporter plasmids (pUC-BENN-CAT, 2× RATS-CAT, 2× mutantRATS-CAT, CMV-CAT) and increasing amounts of pcDNA3-ets-2 (overexpression) and shRNA-ets-2 (knockdown) clones, as indicated. The transfected cells were cultured for 48 h in CM (with 5% FBS). When required, the cells were cultured for an additional 6 h with P/I. They were then processed for CAT analysis using 40 µg of cell lysate per experimental point (27, 39), fluorescence *in situ* hybridization (FISH), or chromatin immunoprecipitation (ChIP) experiments, as indicated.

### Immuno-DNA FISH

A DNA FISH probe was constructed by nick translation using biotin-modified nucleotides. The labeled probe (for sequences, see ChIP Assays, RATS region of HIV-1-LTR) was ethanol precipitated and resuspended in a buffer containing 2× SSC, 50% formamide, and 10% dextran sulfate. Immuno-DNA FISH was performed according to Ref. (41). For each experiment, Jurkat cells transfected with the 2× RATS-CAT or 2× mutantRATS-CAT plasmids were attached on slides, fixed in 4% PFA for 10 min at RT and then washed two times with PBS. The cells were permeabilized in 0.3% Triton X-100 for 4 min at RT, washed two times with PBS, and incubated with a blocking buffer (PBS with 10% FBS and 3% BSA) for 1 h at RT. Next, the cells were incubated with a rabbit polyclonal anti-Ets-2 Ab (Santa Cruz, sc-351) for 1 h at RT, washed three times with PBS with 0.05% Tween-20, and incubated with an Alexa Fluor 488-conjugated donkey anti-rabbit Ab (Thermo Fisher, A-21206) for 1 h at RT. The slides were washed three times with 0.05% Tween-20/PBS and fixed with 4% PFA for 10 min at RT, followed by further permeabilization in 0.3% Triton X-100/PBS for 4 min. For DNA FISH, the slides were washed two times in PBS and incubated in 2× SSC for 5 min. The cells were denatured in 70% formamide, 2× SSC for 3 min at 73°C and dehydrated in ice-cold 70, 85, and 100% ethanol (2 min each step). The cells were air-dried and hybridized O/N in a humidified chamber at 42°C in 15 µl of a hybridization buffer (10% dextran sulfate, 50% formamide, and 2× SSC) combined with 100 ng of DNA FISH probe freshly denatured for 5 min at 75°C and cooled on ice. The slides were then washed two times with 50% formamide/2× SSC, 2× SSC, and 0.01% Tween-20/2× SSC for 5 min and incubated in blocking buffer (10% FBS and 3% BSA/PBS) for 1 h at RT. The cells were then incubated in Streptavidin-Alexa 568 (Life Technologies) solution (diluted in blocking buffer) for 1 h at RT and washed three times with 0.05% Tween-20/PBS. The cells were counter stained with DAPI (1 µg/ml) for 10 min, mounted in MOWIOL, and imaged on a NIKON Eclipse TE 2000-U (wide field). The images were processed with the software FIJI.

### ChIP Assays

For ChIP assays, Jurkat cells (10^7^ cells per experimental point) were transfected with the pUC-BENN-CAT plasmid and cultured in CM ± P/I. The cells were fixed in 1.1% formaldehyde for 10 min followed by quenching with 125 mM glycine for 5 min. They were then lysed and sonicated to generate 200–500 bp DNA fragments. ChIP assays were performed as described (39). Briefly, the reactions were performed in 1 ml sample tubes, using 10 µg of isolated chromatin supplemented with 40 µl of Protein G Dynabeads (Invitrogen) and 5 µg of the appropriate Ab for each ChIP reaction. The Abs used were as follows: anti-Ets-2 (sc-351), anti-POLII (sc-9001), and anti-IgG (sc-2027) as a negative control (all from Santa Cruz). Immunoprecipitated DNA for specific sites was analyzed by real-time PCR using the KAPA SYBR FAST qPCR Kit (Kapa Biosystems) and the Mx3000P_TM_ Quantitative PCR System thermal cycler (Stratagene). The sequences of the ChIP primers used for different genomic regions in real-time PCR were as follows: for the RATS region of HIV-1-LTR, 5′-CCAGAGAAGTTAGAAGAAGCC-3′, 5′-AAGCTTTATTGAGGCTTAAGC-3′, yielding a 378 PCR product and for the TATA region of the β2-m promoter: 5′-CGCCGATGTACAGACAGCAAA-3′, 5′-TGCTGTCAGCTTCAGGAATG-3′, yielding a 230bp PCR product. The optimized PCR conditions were 95°C for 10 min, followed by 40 cycles of 95°C for 30 s and 60°C for 30 s. The results represent the DNA enrichment as the percentage of immunoprecipitated chromatin for every condition and set of primers relative to corresponding chromatin input [100*((Ct IP/Ct INPUT) − (Ct IgG/Ct INPUT)) where Ct is the cycle at which the threshold line is crossed].

### Statistical Analysis

Data are expressed as the mean values (SD or SE) from three independent experiments. Statistical probabilities were evaluated by the Student’s *t*-test or one-way ANOVA. The statistical significance level was set at *p* < 0.05. Data analysis and graphic representation was performed using the GraphPad Prism 5.0 Software.

## Results

### Ets-2 Expression in PBMC Populations

**Figure 1:**
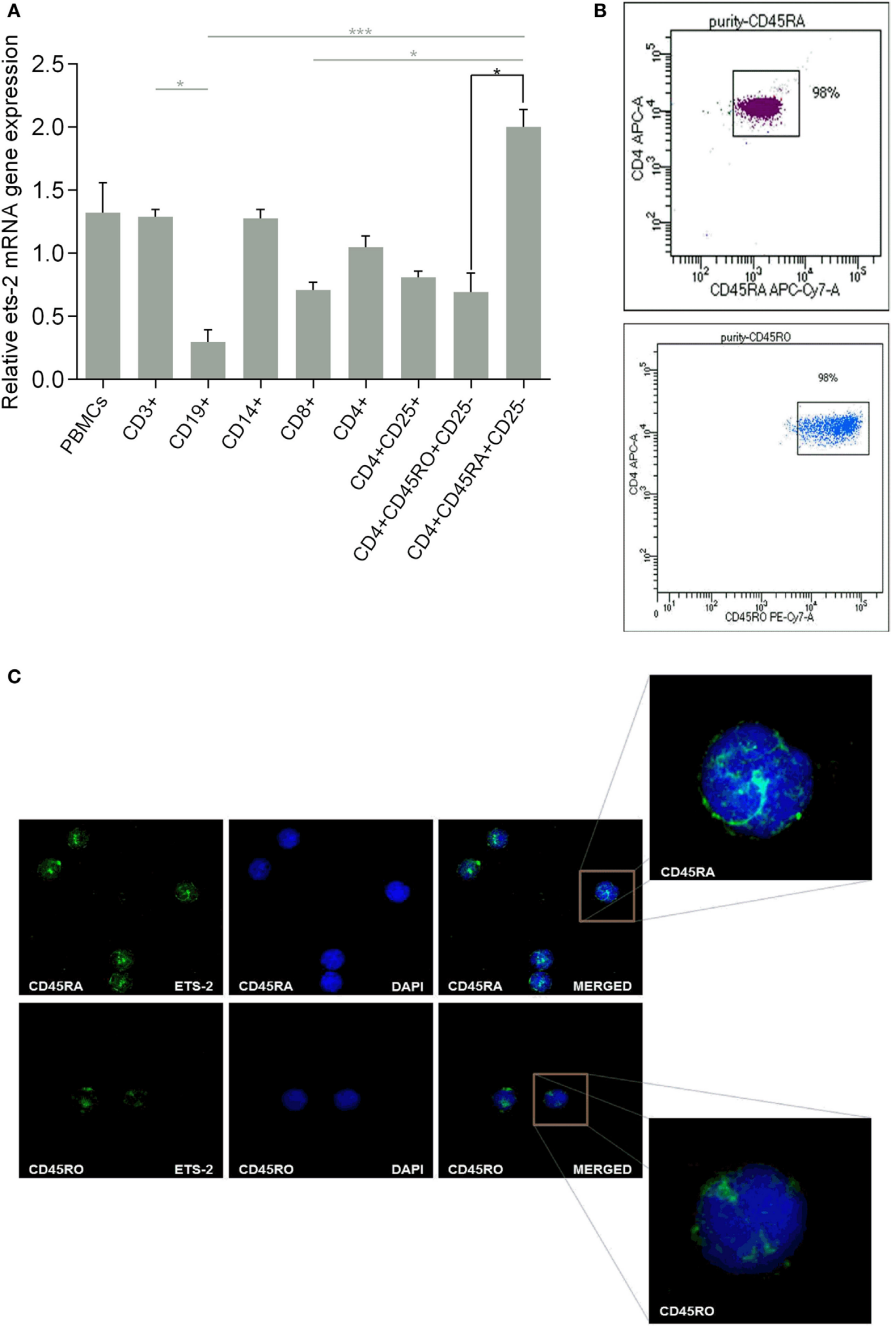
Ets-2 expression in human peripheral blood mononuclear cells (PBMCs); differential expression in naive vs resting memory T helper (Th) cells. **(A)** Relative Ets-2 mRNA gene expression levels measured by real-time PCR in PBMCs and populations thereof. CD3+ T cells, CD19+ B cells, CD14+ monocytes, CD8+ T cytotoxic cells, CD4+ CD25− Th cells, CD4+ CD25+ Th cells (including Tregs), CD4+ CD45RO+ CD25− memory Th cells, and CD4+ CD45RA+ CD25− naive Th cells were isolated by cell sorting from healthy young adults. β2-m served as the normalizer gene for relative expression. The results are presented as the mean values (SD) from three independent experiments. Statistically significant differences are indicated by asterisks (*p < 0.05, ***p < 0.001, one-way ANOVA, more than two groups, gray lines; *p < 0.05, Student’s *t*-test, two groups, black lines). **(B)** Characteristic dot plots showing cell purity after sorting CD4+ CD45RA+ CD25− naive and CD4+ CD45RO+ CD25− memory Th cells. **(C)** Relative amounts of Ets-2 protein in the nuclei of naive and memory Th cells shown by immunofluorescence. The cells were stained for the Ets-2 protein (green), and the nuclei were counterstained with 4,6-diamidino-2-phenylindole (DAPI) (blue). Images are from a Leica TCSSP5 confocal microscope, 63× objective. The results shown are representative of at least three independent experiments.

We investigated the endogenous Ets-2 mRNA levels by real-time PCR in sorted PBMC populations. As shown in Figure 1A, all PBMC populations expressed Ets-2, with naive Th cells expressing the highest levels and B cells the lowest. Focusing on naive and resting memory Th cells, Ets-2 mRNA levels were significantly higher in naive Th cells.

The difference in Ets-2 mRNA levels between naive and resting memory Th cells is also depicted at the protein level. Highly purified naive and resting memory Th cells (Figure 1B) were studied by confocal microscopy for the presence of Ets-2 protein, after staining with an anti-Ets-2 mAb. As shown in Figure 1C, the concentration of Ets-2 in the cell nucleus was significantly higher in naive Th cells compared with resting memory Th cells.

**Figure 2:**
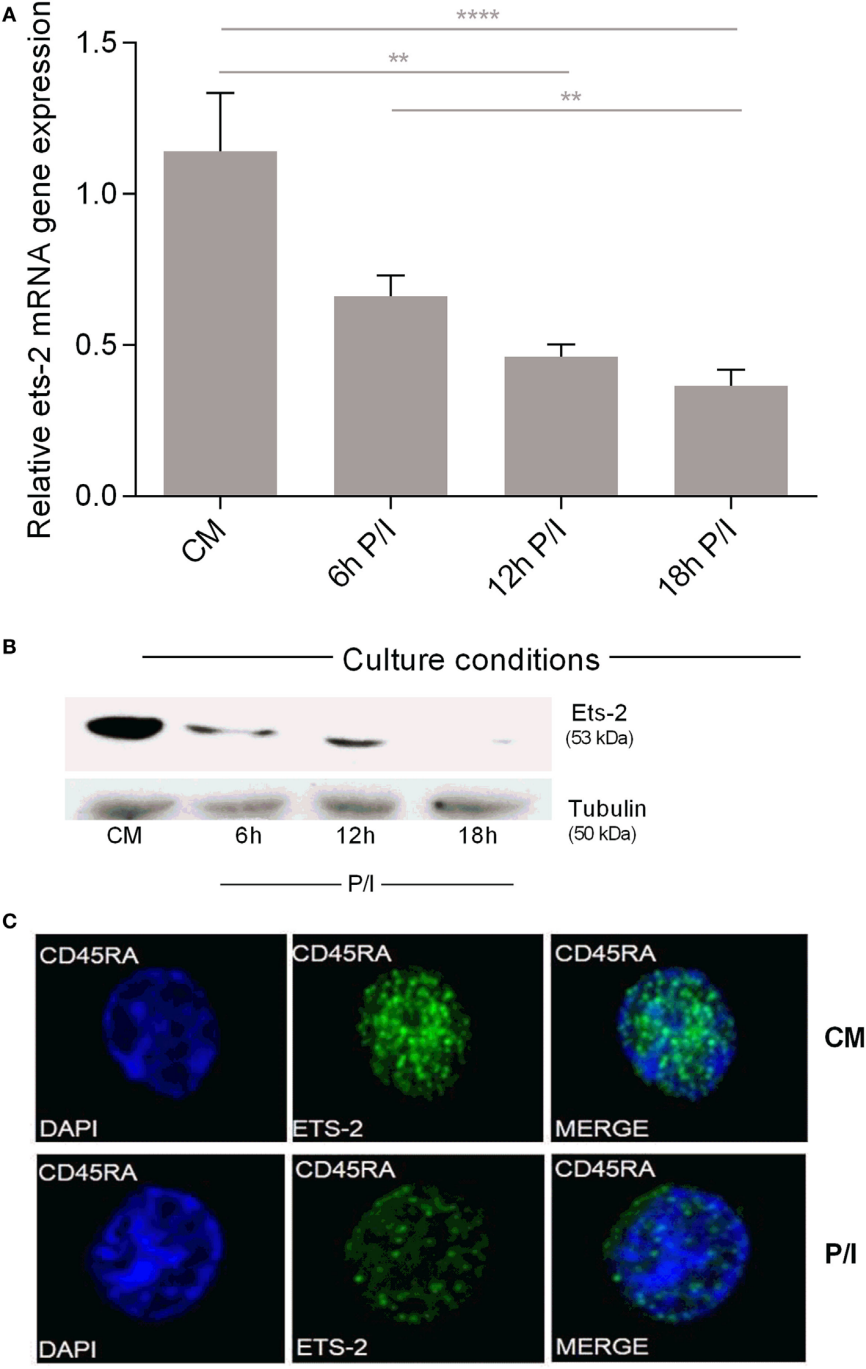
Ets-2 mRNA and protein expression levels in resting vs activated naive T helper (Th) cells. **(A)** Relative Ets-2 mRNA gene expression levels measured by real-time PCR in naive Th cells isolated and cultured in CM or P/I for 6, 12, and 18 h. β2-m served as the normalizer gene for relative expression. The results are presented as the mean values (SD) from three independent experiments. Statistically significant differences are indicated by asterisks (**p < 0.01, ****p < 0.0001, one-way ANOVA). **(B)** Western blot analysis of Ets-2 protein levels in naive Th cells isolated and cultured in CM or P/I for 6, 12, and 18 h. β-Tubulin protein levels were used as an internal control for equal loading. The results shown are representative of at least three independent experiments. **(C)** Relative amounts of Ets-2 protein in the nuclei of resting and 6 h-activated naive Th cells shown by immunofluorescence. The cells were stained for the Ets-2 protein (green), and the nuclei were counterstained with 4,6-diamidino-2-phenylindole (DAPI) (blue). Images are from a Leica TCSSP5 confocal microscope, 63× objective. The results shown are representative of at least three independent experiments.

Measurement of Ets-2 mRNA and protein levels in non-activated and activated naive Th cells showed that the levels of Ets-2 mRNA gradually decreased upon cell activation with P/I (Figure 2A). This reduction was also depicted at the protein level as shown by western blot analysis (Figure 2B) and immunofluorescence (Figure 2C).

These data confirm our earlier findings for Ets-2 expression in PBMC populations purified by a different methodology (39) and show that naive Th cells express Ets-2 mRNA and protein at significantly higher levels compared with resting memory Th cells. The first activation of naive Th cells lowers Ets-2 mRNA and protein to levels that mark a permanent status of Ets-2 expression in resting memory Th cells.

### Endogenous Ets-2 Expression Is Reciprocal to HIV-1-LTR Activation

**Figure 3:**
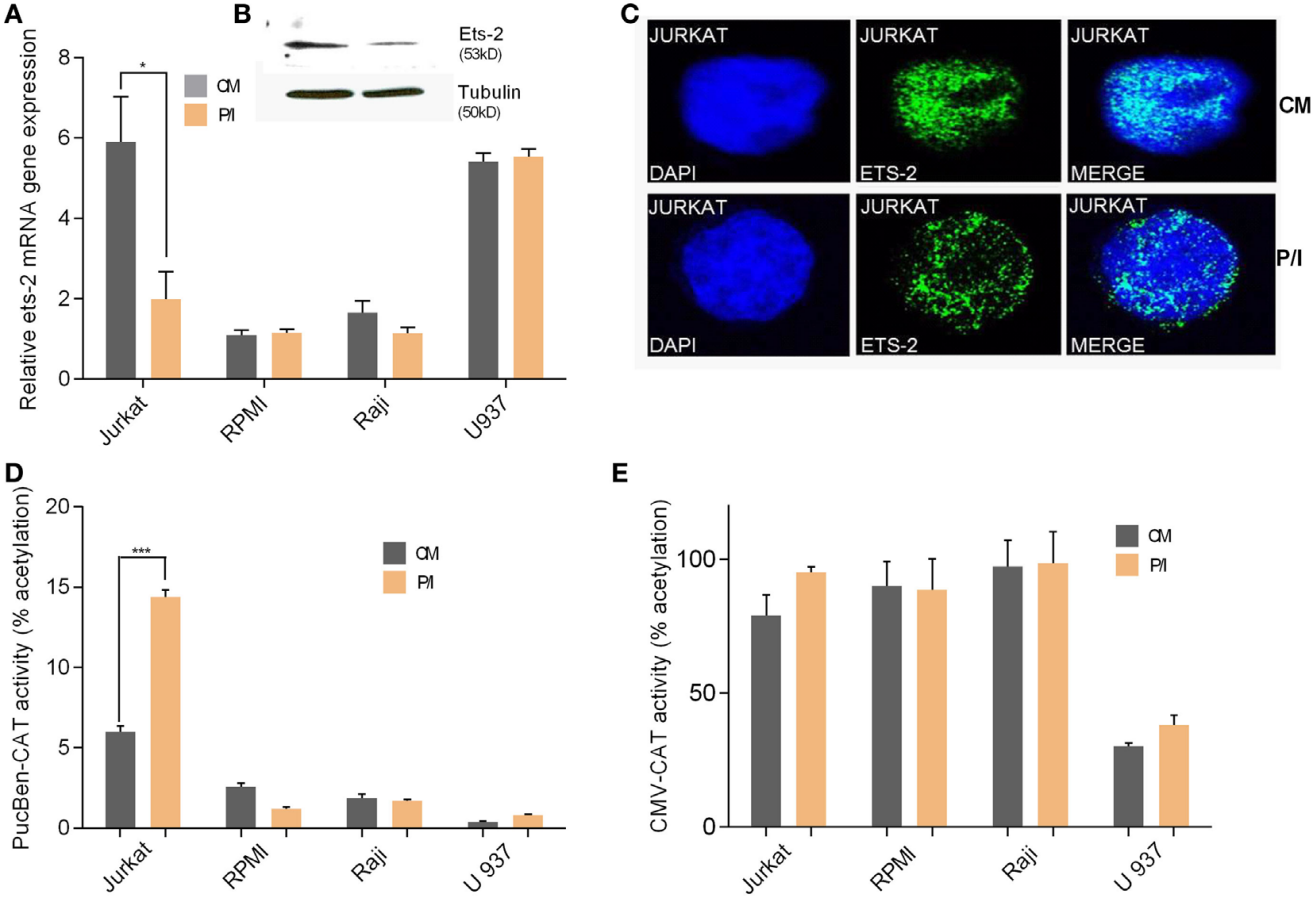
Ets-2 and HIV-1-LTR expression in leukemic cell lines. **(A)** Ets-2 mRNA levels in Jurkat, RPMI1788, Raji, and U937 cells measured by real-time PCR. The cells were cultured in CM or P/I for 6 h. β2-m served as the normalizer gene for relative expression. The results are presented as the mean values (SD) from three independent experiments. Statistically significant differences are indicated by asterisks (*p < 0.05, Student’s *t*-test). **(B)** Western blot analysis of Ets-2 protein levels in Jurkat cells cultured in CM or P/I. β-Tubulin protein levels were used as an internal control for equal loading. The results shown are representative of at least three independent experiments. **(C)** Relative amounts of Ets-2 protein in the nuclei of unstimulated or P/I-stimulated Jurkat cells shown by immunofluorescence. The cells were stained for the Ets-2 protein (green), and the nuclei were counterstained with 4,6-diamidino-2-phenylindole (DAPI) (blue). Images are from a Leica TCSSP5 confocal microscope, 63× objective. The results shown are representative of at least three independent experiments. **(D,E)** Jurkat, RPMI8866, Raji, and U937 cells were transfected with 20 µg of pUC-BENN-CAT or CMV-CAT plasmids and cultured in CM or P/I. CAT assays were performed in cell extracts. The results are presented as the mean values (SD) from three independent experiments. Statistically significant differences are indicated by asterisks (***p < 0.001, Student’s *t*-test).

We investigated the endogenous Ets-2 mRNA levels in Jurkat, RPMI8866, Raji, and U937 cell lines cultured in uninduced (CM) and induced conditions (P/I) (Figure 3A). Jurkat cells expressed high levels of Ets-2 mRNA in CM, and its synthesis was decreased upon stimulation with P/I, whereas Ets-2 expression was not modified in stimulated RPMI8866, Raji, and U937 cells (Figure 3A). The reduction of Ets-2 expression in stimulated Jurkat cells is also depicted at the protein level, shown by western blot analysis (Figure 3B) and immunofluorescence (Figure 3C).

Transfection of Jurkat, RPMI8866, Raji, and U937 cells with the pUC-BENN-CAT plasmid or the CMV-CAT plasmid (as a negative control) showed higher pUC-BENN-CAT activity in unstimulated Jurkat cells compared with RPMI8866, Raji, and U937 cells (Figure 3D). pUC-BENN-CAT activity was increased 2.4× fold in P/I-activated Jurkat cells but remained at the same low levels in P/I-activated RPMI8866, Raji, and U937 cells (Figure 3D). CMV-CAT activity was unaffected by culture conditions in all the cell lines tested (Figure 3E).

These data indicate that in Jurkat cells, there is a negative causality between Ets-2 and HIV-1-LTR expression.

### Ets-2 Represses HIV-1-LTR Activity through the RATS Sequence

**Figure 4:**
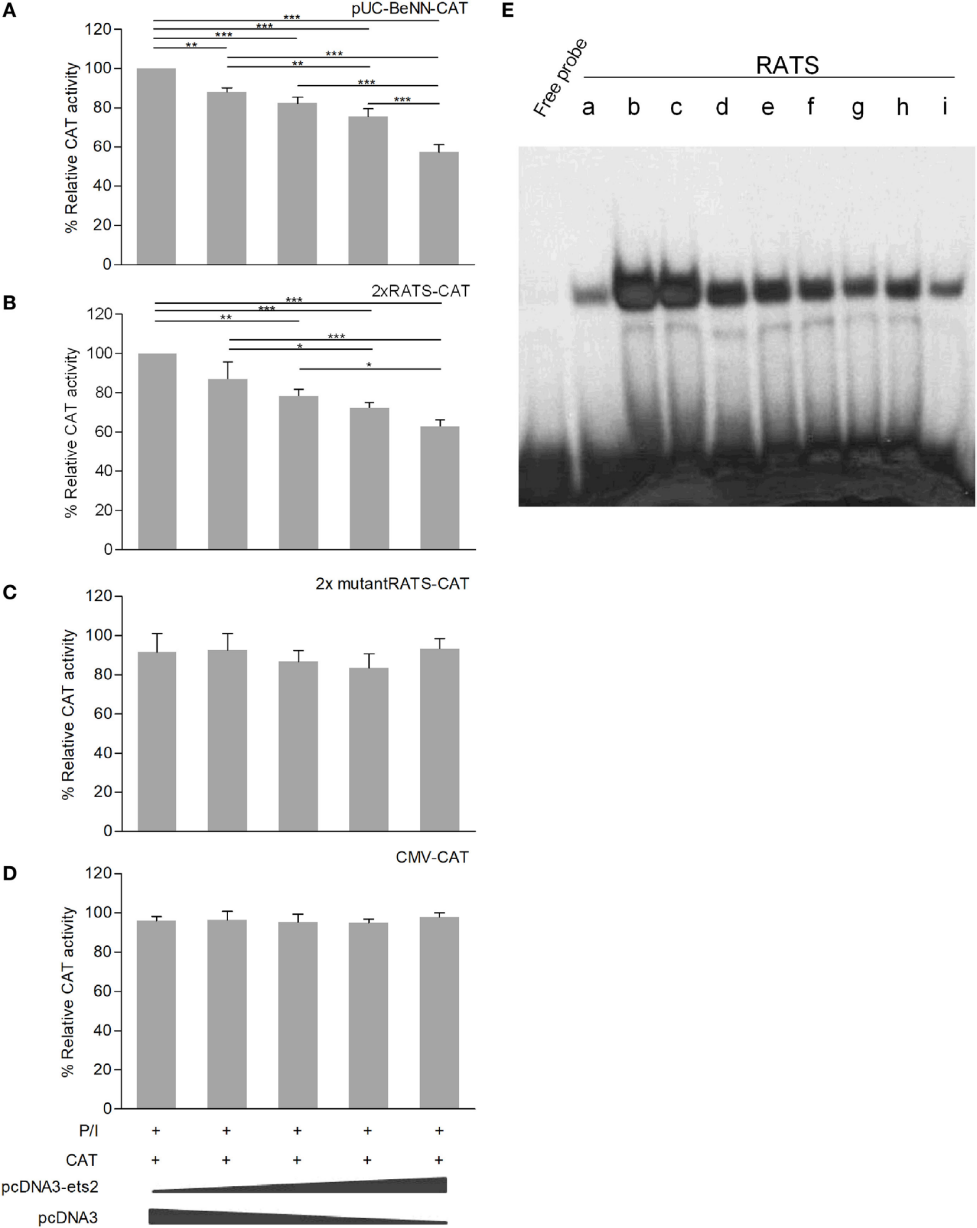
Ets-2 silences HIV-1-LTR expression through binding to the repressor–activator target sequence (RATS) element. Jurkat cells were transfected with increasing amounts of the Ets-2 overexpression plasmid pcDNA3-ets-2 (0, 0.5, 1, 2, and 4 µg) and a constant amount (2 µg) of the reporter plasmids: **(A)** pUC-BENN-CAT, **(B)** 2× RATS-CAT, **(C)** 2× mutantRATS-CAT, or **(D)** CMV-CAT as control. The amount of transfected DNA per sample was retained to 6 µg by the addition of appropriate amounts of vector pcDNA3 DNA. The cells were cultured in P/I. CAT assays were performed in cell extracts. The results are presented as the mean values (SD) from three independent experiments. Statistically significant differences are indicated by asterisks (*p < 0.05, **p < 0.01, ***p < 0.001, one-way ANOVA). **(E)** Ets-2 binding activity to the RATS element. T helper-cell nuclear extracts were passed through a protein A/G agarose column to remove proteins that bind to A/G agarose. The resulting eluate (a) was mixed for 1 h with 20 µg anti-Ets-2 Ab and passed through a protein A/G agarose column. Bound Ets-2 was eluted and collected in consecutive 1 ml aliquots. The consecutive protein aliquots (b–i) were precipitated by PEG8000, and the pellets were redissolved in buffer. Electrophoretic mobility shift assays were performed with the RATS oligonucleotide probe.

Co-transfection of P/I-stimulated Jurkat cells with increasing amounts of the Ets-2 overexpression vector pCDNA3-ets-2 and stable amounts of the reporter plasmids pUC-BENN-CAT, 2× RATS-CAT, 2× mutantRATS-CAT, and CMV-CAT (Figure 4) showed that transfection with increasing amounts of pCDNA3-ets-2 led to a gradual reduction of the pUC-BENN-CAT (Figure 4A) and 2× RATS-CAT (Figure 4B) expression activities. This reduction was not observed when the cells were co-transfected with pCDNA3-ets-2 and 2× mutantRATS-CAT (Figure 4C) or CMV-CAT (Figure 4D).

To verify that Ets-2 protein binds to RATS in naive Th cells, we performed EMSA with purified Ets-2 isolated from nuclear extracts from peripheral blood Th cells. The protein extracts were passed through an Ets-2 Ab-binding column, and EMSAs were performed using an RATS probe mixed with consecutive protein eluates. As shown in Figure 4E, Ets-2 bound to RATS in a dose-dependent manner.

**Figure 5:**
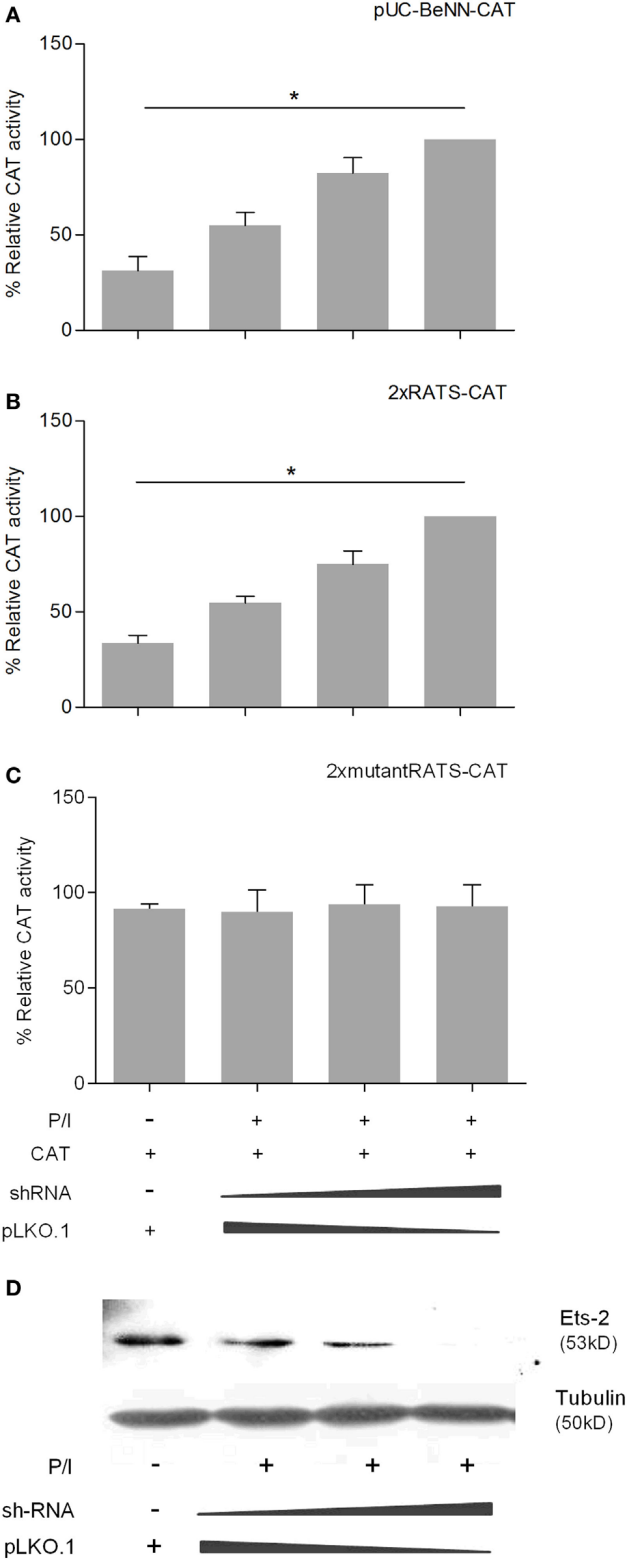
Ets-2 knockdown increases HIV-1-LTR expression. Jurkat cells were transfected with increasing amounts of the Ets-2 knockdown shRNA clones (0, 0.5, and 1 µg) and a constant amount (1 µg) of the reporter plasmids **(A)** pUC-BENN-CAT, **(B)** 2× RATS-CAT, or **(C)** 2× mutantRATS-CAT. The amount of transfected DNA per sample was retained to 2 µg by the addition of appropriate amounts of vector pLKO.1 DNA. The cells were cultured ±P/I as indicated. CAT assays were performed in cell extracts. The results are presented as the mean values (SD) from three independent experiments. Statistically significant differences are indicated by asterisks (*p < 0.05, one-way ANOVA). **(D)** Western blot analysis to verify the silencing of Ets-2 in the transfected cells. β-Tubulin protein levels were used as an internal control for equal loading. The results shown are representative of at least three independent experiments.

To confirm the negative causality between Ets-2 and pUC-BENN-CAT or 2× RATS-CAT expression, we co-transfected Jurkat cells with increasing amounts of ets-2 mRNA knockdown clones and constant amounts of pUC-BENN-CAT, 2× RATS-CAT, or 2× mutantRATS-CAT (Figure 5). The expression of the reporter genes was measured by CAT assays. The results show that Ets-2 knockdown resulted in a gradual increase in pUC-BENN-CAT (Figure 5A) and 2× RATS-CAT activity (Figure 5B) but not in 2× mutantRATS-CAT (Figure 5C). Ets-2 knockdown was confirmed at the protein level by Western blot analysis (Figure 5D).

### *In Vivo* Interaction between Ets-2 Protein and the RATS Sequence

**Figure 6:**
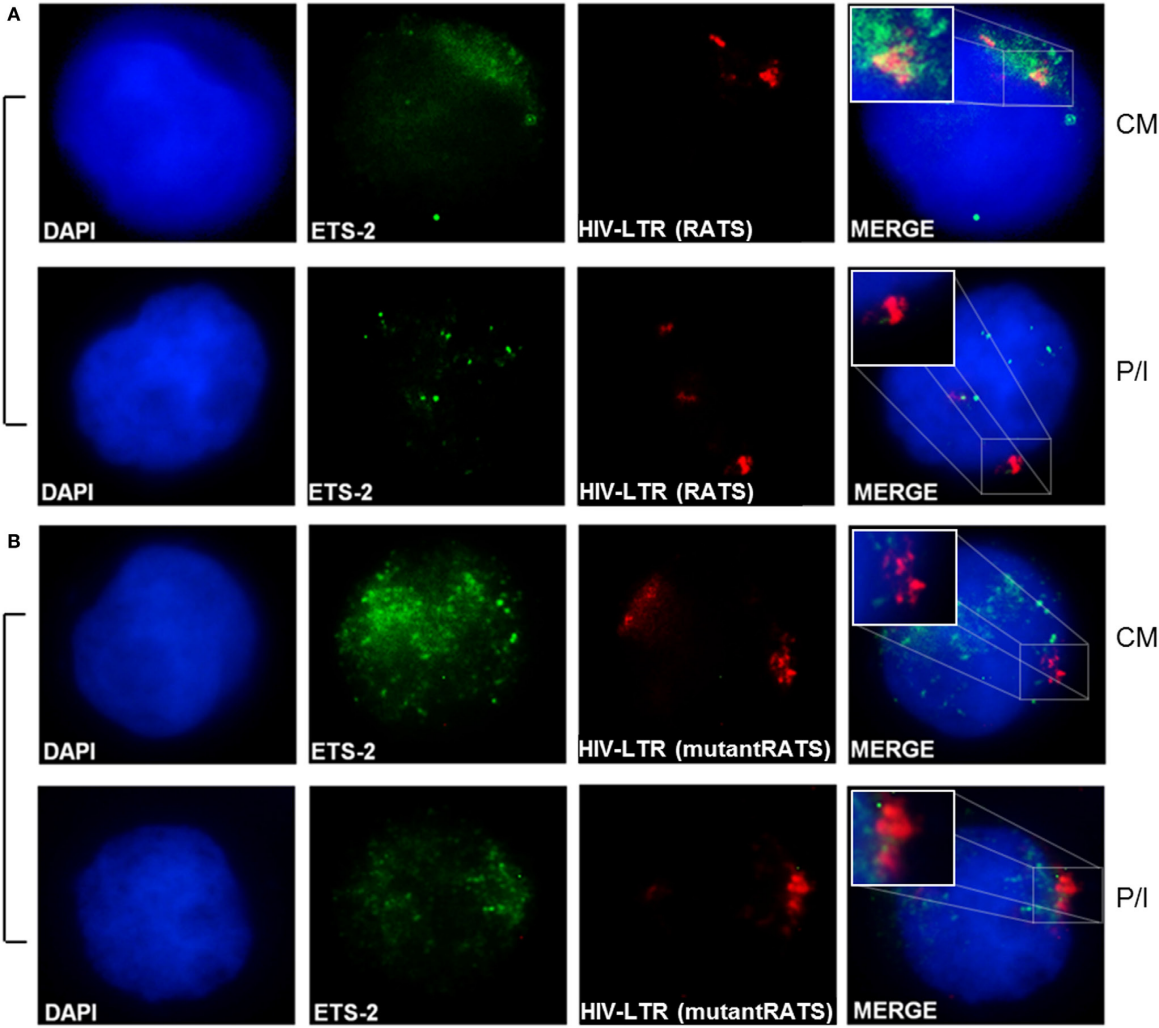
Physical interaction between endogenous Ets-2 protein and the repressor–activator target sequence (RATS) element. Jurkat cells were transfected with the **(A)** 2× RATS-CAT or **(B)** 2× mutantRATS-CAT plasmids. Post-transfection, the cells were cultured in CM or P/I and attached on slides. The cells were stained for the Ets-2 protein (green), and the nuclei were counterstained with 4,6-diamidino-2-phenylindole (DAPI) (blue). Next, the cells were denatured and hybridized with a DNA fluorescence *in situ* hybridization probe that identifies and attaches to the RATS region of HIV-1-LTR (red). Yellow spots indicate Ets-2/RATS co-localization [**(A)**, CM]. Images are from a NIKON Eclipse TE 2000-U microscope, 100× objective. The results shown are representative of at least three independent experiments.

To confirm the physical interaction between the endogenous Ets-2 protein and the RATS sequence of HIV-1-LTR, Immuno-DNA-FISH experiments were performed (Figure 6). Jurkat cells were transfected with 2× RATS-CAT or 2× mutantRATS-CAT plasmids and cultured for 48 h in CM. When indicated, the cells were cultured for additional 6 h in the presence of P/I. In CM conditions, Ets-2 protein co-localized with the RATS sequence in Jurkat cells transfected with the 2× RATS-CAT plasmid, whereas in P/I-activated cells the Ets-2/RATS co-localization was not observed (Figure 6A). Conversely, when Jurkat cells were transfected with the 2× mutantRATS plasmid no co-localization was observed under CM of P/I conditions (Figure 6B).

**Figure 7:**
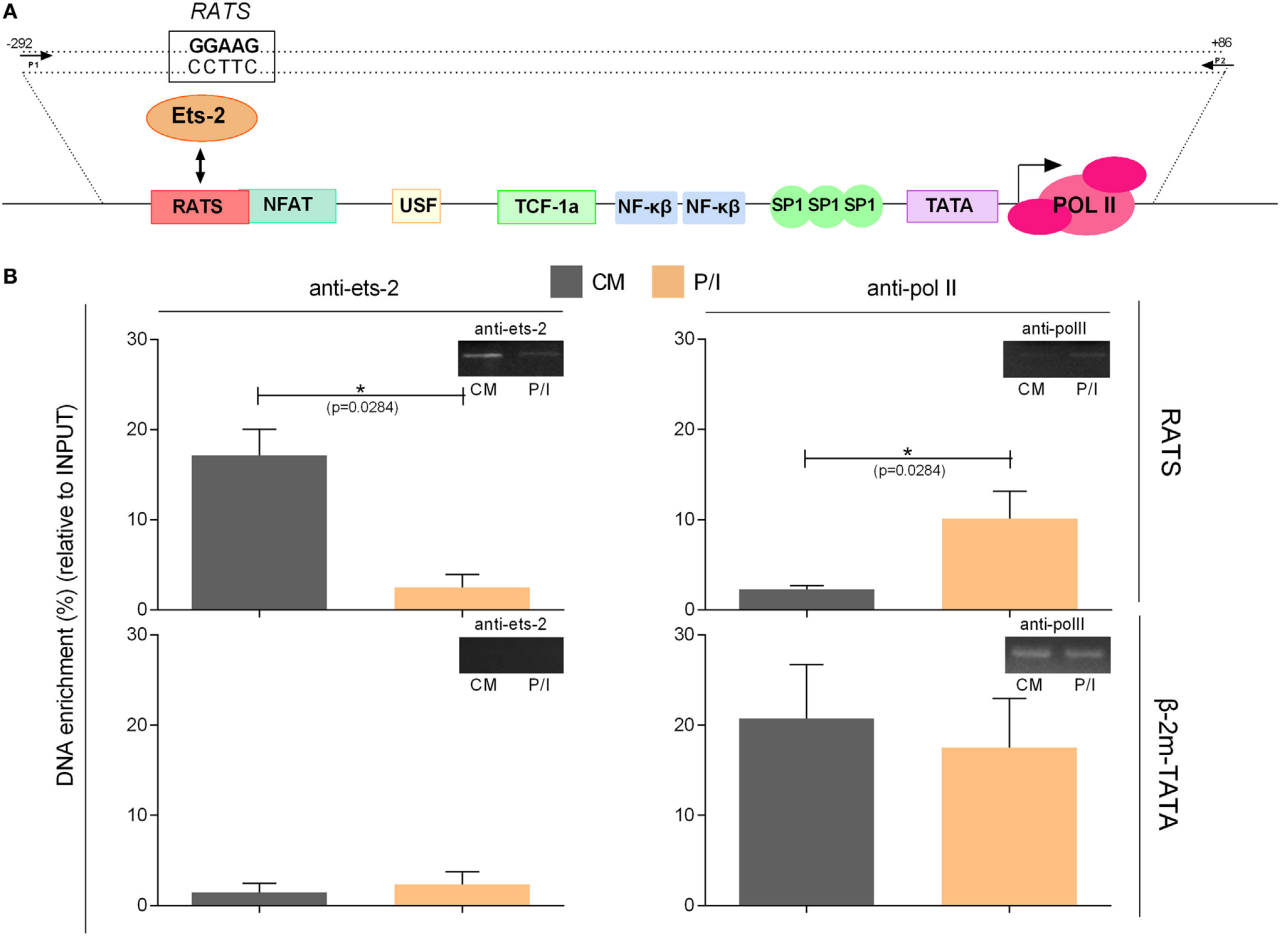
Chromatin immunoprecipitation (ChIP) assays to verify the specificity of the *in vivo* binding of Ets-2 to repressor–activator target sequence (RATS). **(A)** Schematic representation of the HIV-1-LTR sequence showing the relative binding positions of transcription factors, the TATA box, and the transcriptional initiation start site (+1). The arrows indicate the relative position of the primers pair used for the detection, by real-time PCR, of the HIV-1-LTR region that encompasses the RATS element and the virus TATA box from the immunoprecipitated chromatin. **(B)** Jurkat cells were transfected with the pUC-BENN-CAT plasmid and were cultured in CM ± P/I for 6 h. The cells were harvested, and their extracts were prepared for ChIP analysis. ChIP assays were carried out with Abs to Ets-2, POLII (and IgG for background determination). The results represent the DNA enrichment as the percentage of immunoprecipitated chromatin for every condition and set of primers relative to the corresponding chromatin input. To control for the accuracy of the ChIP assays, for the specificity of the Abs and the amplified regions, real-time PCR was performed to detect the TATA region of the β2-m gene promoter (β2-m-TATA). The results shown are the mean values (SE) of three independent experiments. Statistically significant differences are indicated by asterisks (*p < 0.05, Student’s *t*-test). The control antibody to IgG failed to precipitate chromatin.

To confirm the specificity of the *in vivo* Ets-2 protein binding to RATS, ChIP experiments were performed from chromatin isolated from Jurkat cells transfected with pUC-BENN-CAT and cultured for 48 h in CM or for an additional 6 h in the presence of P/I (Figure 7). Cross-linked chromatin was subjected to immunoprecipitation using Abs against human Ets-2 and POLII or a negative control Ab to IgG. The relative positions of Ets-2 and POLII on HIV-1 LTR are shown in Figure 7A. A chromatin region that encompasses the human β-2m gene promoter including the TATA element was used as a negative control for Ets-2 binding. Ets-2 or POLII binding to the RATS or the β-2m-TATA sequences was detected by real-time PCR using specific sets of primers. In CM conditions, Ets-2 binding to the RATS sequence was significantly higher compared with P/I conditions (Figure 7B, anti-ets-2, RATS). The inability to detect Ets-2 binding on the RATS sequence in activated cells is consistent with the results from the Immuno-DNA-FISH (Figure 6A). P/I induction of the cells resulted in the engagement of POLII to the LTR sequence that acts as the virus promoter, a fact consistent with the transcriptional activation of the virus (Figure 7B, anti-polII, RATS). No Ets-2 binding to the β-2m gene promoter was observed under CM or P/I conditions (Figure 7B, anti-ets-2, β-2m-TATA); by contrast, the anti-POLII Ab bound to the genomic region around the TATA box of β-2m promoter under both CM and P/I conditions (Figure 7B, anti-polII, β-2m-TATA).

The data confirm that Ets-2 binds to the RATS element of the HIV-1 LTR *in vivo* in unstimulated cells. Ets-2 binding is significantly reduced when the cells are activated, a state in which the expression of HIV-1 LTR is significantly increased (Figure 3D).

## Discussion

Naive Th lymphocytes are one of the main cellular reservoirs of the HIV-1 virus in infected individuals (6); therefore, the involvement of host transcription factors of infected naive Th cells in HIV-1 suppression, through their binding to the virus LTR, constitutes a major cause of viral latency in these cells. Our previous studies have demonstrated that in naive, but not activated or memory Th cells, a transcription factor binds to the RATS element of HIV-1-LTR and represses viral expression (27). The RATS sequence shares significant homology with the ARRE-2 sequence in the IL-2 promoter, where the transcription factor Ets-2 binds and suppresses IL-2 expression in naive Th cells (39).

Our results demonstrate a significant inhibitory potential for Ets-2 that binds to the RATS element of HIV-1 LTR and represses the expression of LTR-driven reporter genes and, also, that the actual amount of Ets-2 protein plays a critical role in its repressive activity.

Our results fit well in the current understanding of the role of the transcriptional factors of the Ets family in controlling the expression and the differentiation of a wide variety of genes in hemopoietic cells. Ets factors bind to a DNA region spanning 12–15 bp containing a central GGA motif (32), present in both the IL-2 promoter and HIV-1 LTR. Ets proteins are expressed during development of the immune system, and a comprehensive analysis of the expression of members of this family revealed striking dynamic patterns in all cells tested (35). Identification of Ets-2 as a HIV-1 LTR repressor in naive Th cells could explain certain aspects of the immunopathogenesis of HIV infection. On one hand, the discovery of a cell type-specific repressor could provide an explanation as to why the virus stays latent in these cells, in spite of its capacity to infect them. Being in a state of transcriptional silence, the virus does not express the enzymes that are responsible for replication of HIV genome and therefore is not vulnerable to the antiretroviral agents currently used to treat the disease (42). Hence, naive Th cells constitute a viral reservoir that underlies the latency of the infection, resistance to treatment and decline of the repertoire of antigenic responses seen during disease progression.

Competition between the viral LTR and endogenous cellular promoter targets for Ets-2 could provide a unifying mechanism behind the autoimmune phenomena observed during HIV infection, present in 1–60% of all infected patients (43–48), as well as the role of natural infections in enhancing viral replication (49–52). Binding of Ets-2 to the retroviral promoter would result in decreased availability of the former to inhibit its endogenous targets, which would make the cells harboring the virus hyperresponsive to antigenic stimuli. In such a population of cells, a weaker antigenic stimulation (i.e., one that comes from ubiquitous self-antigens) might provide a stimulus strong enough to trigger an (auto)-immune response. Antigens in the setting of community-acquired infections (i.e., viral, bacterial, mycobacterial, and parasitic) may play a similar role. Evidence to support such an interaction comes from experimental data examining the influence of HIV infection on the expression of reporter genes driven by either the IL-2 promoter or tandem copies of the ARRE-2 site of the IL-2 promoter in transfected T-cell clones (53). In that system, augmentation of the IL-2 promoter-driven reporter-gene activity was up to 155× fold higher when cells were activated in the presence of HIV virions. In the light of our earlier study (27) and the data we presented in this work, we can propose that the RATS element on HIV-1 LTR constitutes a more efficient competitor for the repressor molecule Ets-2 in the host cell. The reduced availability of repressor molecules to cover its endogenous target may lead to the activation of naive Th cells by weaker antigenic stimuli. The net outcome would be a paradoxical state of autoimmunity accompanied by worsening immunosuppression as the functional repertoire of T cells is progressively depleted. This mechanism could account for the simultaneous occurrence of autoimmunity and immunosuppression in the setting of the HIV infection progression toward clinical AIDS, as well as the reemergence of autoimmunity after immune reconstitution has been effected by HAART (54).

The functional involvement of Ets-2 in autoimmune phenomena is also suggested by EMSA experiments using nuclear extracts of T cells from autoimmune patients, which demonstrated the absence of ARRE-2 binding activity in naive Th cells [(55), and our unpublished data] during both disease activity and remission, suggesting that this abnormality is primary, rather than an epiphenomenon. During lymphocyte maturation, a failure in the acquisition of the repressor by some otherwise mature thymocytes could result in an increased number of peripheral naive Th cells, which are more prone to develop into autoreactive clones upon antigenic stimulation. Such an epigenetic abnormality can be potentially mirrored in naive Th-cell infection by HIV-1.

Recent studies have demonstrated decreased IL-2 expression in primary Th cells with latent HIV-1 infection even after activation of the cells with mitogens (56, 57). On the other hand, in HIV-1 elite controllers that are characterized by undetected viral load (<50 HIV-1 RNA copies/ml) and normal Th-cell numbers, high levels of IL-2 are detected in their blood serum compared with progressors (58). To note, IL-2 plays an important role in HIV infection, since it contributes to Th-cell proliferation and their protection from apoptosis (59). It would be interesting to investigate whether Ets-2 expression levels in naive Th cells affect the progression of HIV-1 infection in progressors vs elite controllers.

Our earlier EMSA experiments performed with nuclear proteins isolated from resting peripheral blood T cells that exhibited repressor activity on IL-2 and HIV-1 expression, and, as probes, either the ARRE-2 element of the IL-2 promoter or the RATS element of the HIV-1-LTR, showed the formation of similar complexes. Cross-competition experiments with varying quantities of ARRE-2 and RATS cold probes showed that the RATS element competed much more efficiently for protein binding to ARRE-2 compared with ARRE-2 competition for protein binding to RATS. Specifically, 50-fold of cold RATS probe was sufficient to compete for protein binding to ARRE-2 to a level attained with 1,000-fold of cold ARRE-2 probe to compete for protein binding to RATS (27). These early observations, taken together with our recent discoveries that this repressor protein that binds to ARRE-2 and RATS in naive Th cells is the transcription factor Ets-2 [Ref. (29, 39) and this work], indicate that Ets-2 has the propensity to preferentially target the HIV-1 LTR during infection, thus contributing to viral latency and maintenance of viral reservoirs in patients, despite long-term therapy.

Although it will be considerably difficult to perform these direct competition experiments in naive Th cells infected with latent HIV-1 because they are extremely rare in peripheral blood (60), our results that show a similar pattern of Ets-2 expression between peripheral blood naive Th cells isolated from immunocompetent HIV-1+ patients and healthy controls (pages 4–7 and Figure S3 in Supplementary Material) confirm that Ets-2 is available and can act as a repressor if and when the patients’ naive Th cells become infected.

The identification of Ets-2 as transcriptional silencer of the HIV-1 virus sets the stage for investigations into the immunopathogenesis of the HIV-1 infection in the domain of genomic conflict.

## Ethics Statement

This study was carried out in accordance with the recommendations of Patras University Hospital Ethics and Scientific Committees with written informed consent from all subjects. All subjects gave written informed consent in accordance with the Declaration of Helsinki. The protocol was approved by Patras University Hospital Ethics and Scientific Committees.

## Author Contributions

AM and AS conceived and coordinated the study; IP, FK, and IA performed the experiments, analyzed the data, and prepared the figures; TG and CA advised on methodology; KA and CG were responsible for obtaining the written informed consent from the HIV-1+ patients, collecting the patients’ blood samples, and providing the patients’ clinical/laboratory data; IP, TG, CA, and AM wrote the manuscript.

## Conflict of Interest Statement

The authors declare that the research was conducted in the absence of any commercial or financial relationships that could be construed as a potential conflict of interest.
