# Supplementary material for: Ets-2 Acts As a Transcriptional Repressor of the Human Immunodeficiency Virus Type 1 through Binding to a Repressor–Activator Target Sequence of 5′-LTR
Source: Front Immunol. 2018 Jan 4;8:1924. doi: 10.3389/fimmu.2017.01924 (PMC5758550; doi:10.3389/fimmu.2017.01924)
Supplement: Supplementary file 1 [file data_sheet_1.PDF]

Supplementary Material

Ets-2 acts as a transcriptional repressor of the human immunodeficiency virus type 1 through binding to a repressor-activator target sequence of 5'-LTR

Ioannis Panagoulias, Fotios Karagiannis, Ioanna Aggeletopoulou, Tassos Georgakopoulos, Christos P. Argyropoulos, Karolina Akinosoglou, Charalambos Gogos, Athanasios Skoutelis and Athanasia Mouzaki

*** Correspondence:** Athanasia Mouzaki: mouzaki@upatras.gr

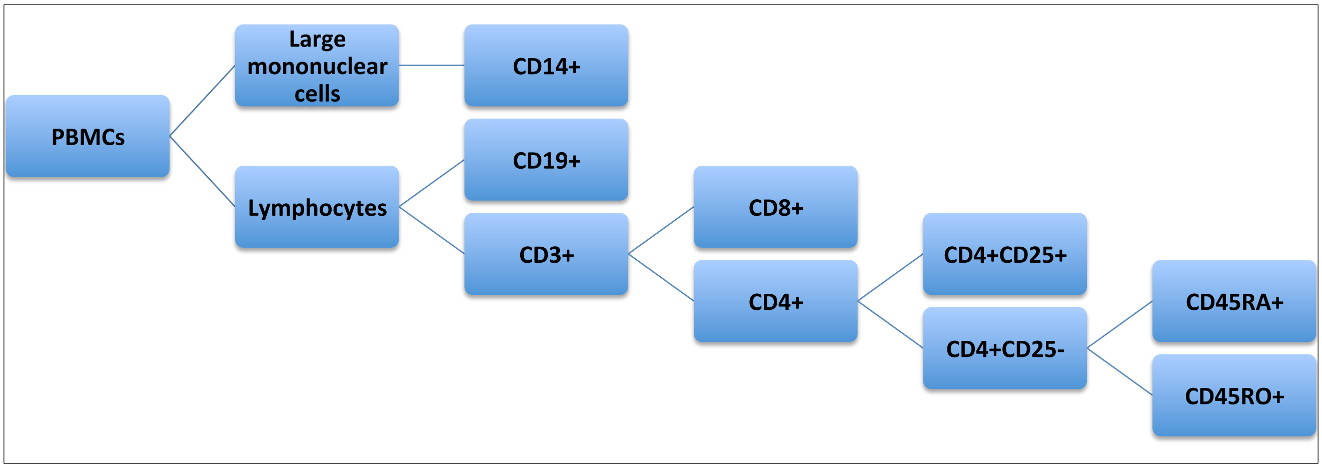

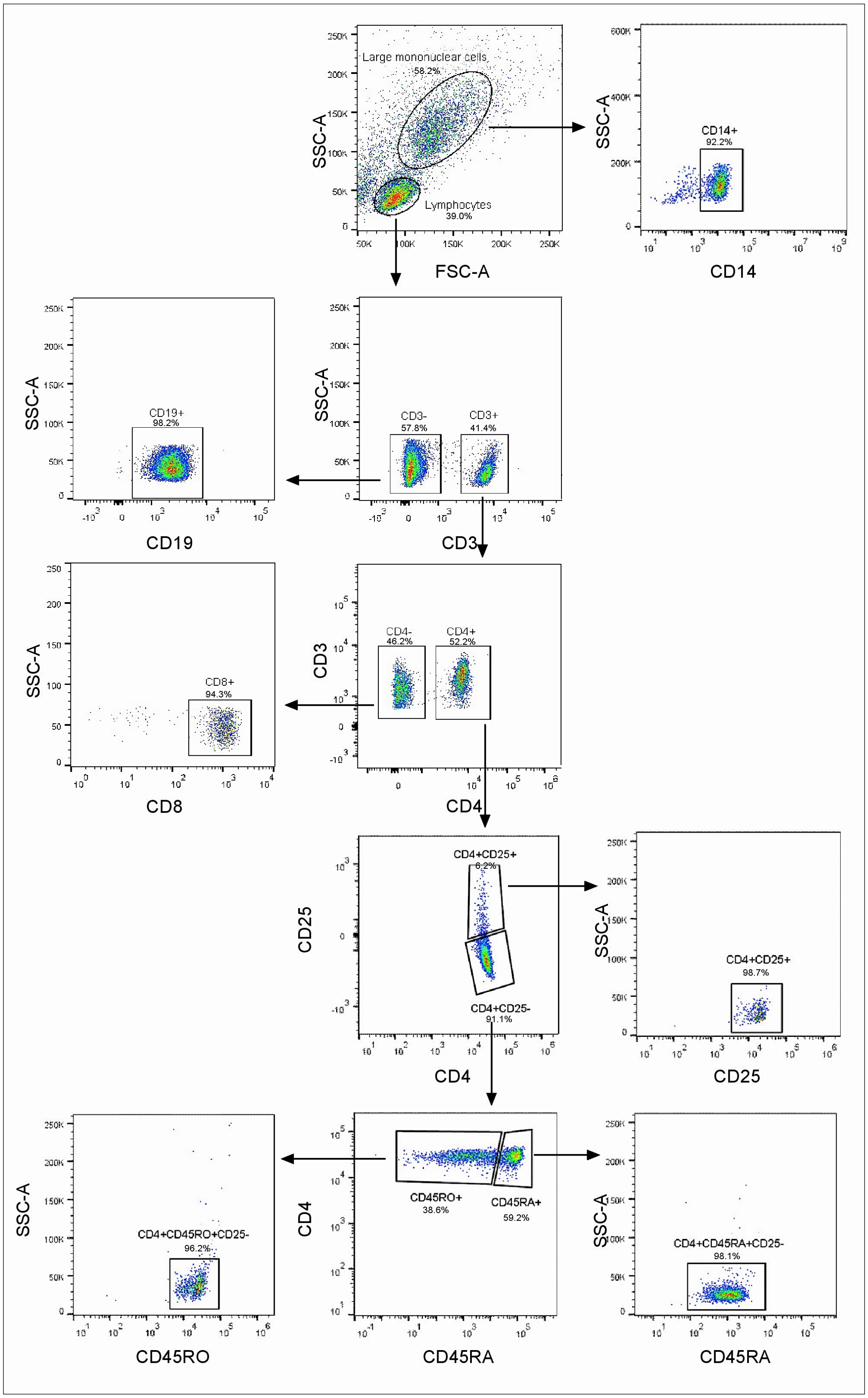

Supplementary Figure 1. Sorting strategy for the isolation of the peripheral blood mononuclear populations (PBMCs) used in this study. Examples are given of the phenotyping of the sorted cell populations to demonstrate their purity.

Determination of CD45RA surface expression on Th cells before and after P/I stimulation

Peripheral blood samples (3 ml) from 10 healthy young adults (6F/3M, age range 22-35 years) were collected in heparinized tubes. PBMCs were isolated and cultured for 6h in the absence or presence of the mitogens ionomycin and phorbol myristate acetate (P/I), as described in the M&M of the manuscript.

At the end of the culture period, the cells where washed with PBS and phenotyped, using the mouse anti-human mAbs CD4-APC (clone RPA-T4), CD45RA-APC-H7 (clone 5H9) and CD45RO-PE-Cy7 (clone UCHL1) (BD Biosciences). Flow cytometric acquisition and analysis were performed on at least 10,000 acquired events per sample, using a Coulter EPICS-XL-MCL cytometer. The data were analyzed using the FlowJo V7.5 software (Tree Star Inc.).

The results (Supplementary Figure 2) show that naive Th cells retain the CD45RA marker after 6h stimulation with P/I. Memory Th cells also retain the CD45RO marker after 6h stimulation with P/I.

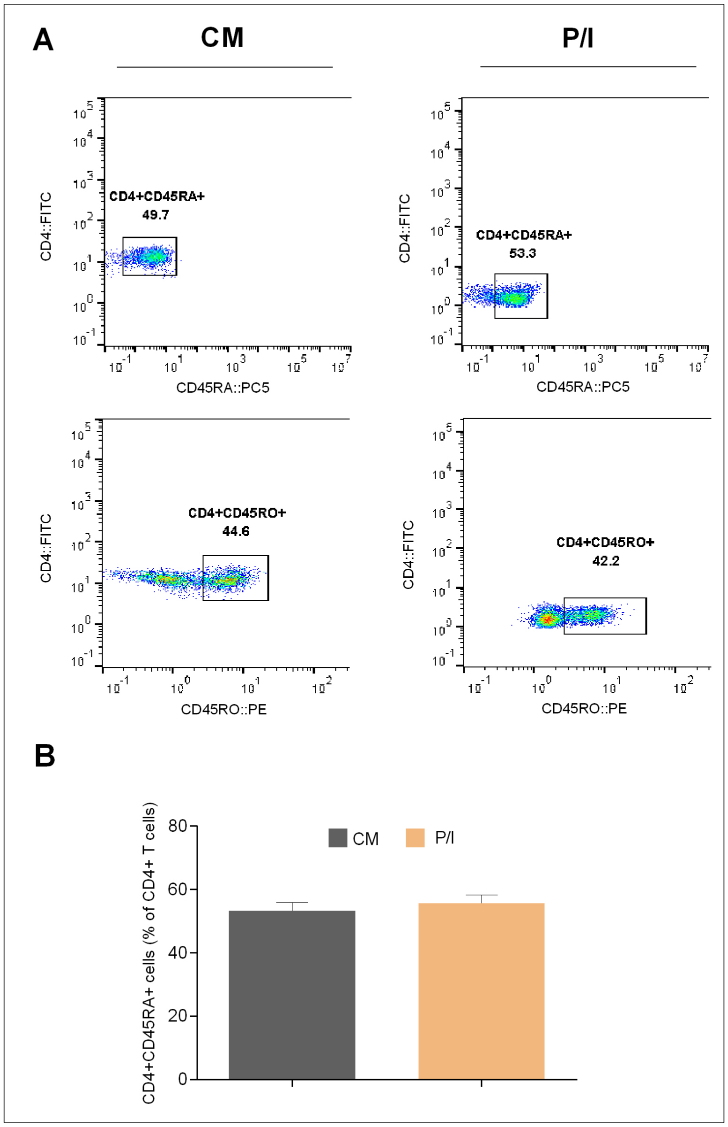

Supplementary Figure 2. Flow cytometric analysis to determine the effect of P/I stimulation on the CD45RA marker on the surface of Th cells. (**A**) A representative analysis is shown for CD45RA+ naive and CD45RO+ memory CD4+ T-cells before and after 6h stimulation with P/I. (**B**) The results (mean with SD) of the analysis of CD45RA+ naive Th cells from 10 healthy individuals. CM, culture medium; P/I, culture medium with P/I.

Determination of Ets-2 expression in naive Th cells of HIV+ patients

Ten consecutive HIV-1+ patients (Supplementary Table 1) with scheduled appointments to the outpatient clinic of the Infectious Diseases Unit of Patras University Hospital gave samples of their peripheral blood for the study. All subjects gave written informed consent. The study protocol is covered by an approved protocol by the Patras University Hospital Ethics (Re: 358/06.07.2017) and Scientific (Re: 17759/26.07.2017) Committees for the study of the role of the transcription factor Ets-2 in the pathogenesis of HIV-1. The Hospital abides by the Helsinki declaration on ethical principles for medical research involving human subjects.

All patients presented in good clinical condition without any complains or findings at the time of enrollment except for oral candidiasis and dermatitis in two patients (P2 and P4, Suppl. Table 1). All patients except for patient P4 - who was about to begin -were already on antiretroviral treatment according to standard guidelines (refs. S1, S2), presented no adverse events, were in good compliance with medication and were on a regular follow up visit schedule.

Supplementary Table 1. Data of HIV+ patients

Patient	Gender	Age (years)	CD4+ T-cell count (/μl)	Year of diagnosis	Current treatment regimen	Clinical condition
P1	M	81	546	2003	Dolutegravir, Abacavir, Lamivudine	Good clinical condition upon assessment
P2	M	35	359	2000	Emtricitabine, Tenofovir disoproxil fumarate, Darunavir, Raltegravir, Ritonavir	Good clinical condition upon assessment, except for oral candidiasis
P3	M	37	576	2000	Dolutegravir, Abacavir, Lamivudine	Good clinical condition upon assessment
P4	M	53	360	2004	Naive	Good clinical condition upon assessment, except for dermatitis
P5	M	43	891	2010	Emtricitabine, Tenofovir disoproxil fumarate, Atazanavir, Ritonavir	Good clinical condition upon assessment
P6	M	62	491	2007	Elvitegravir, Cobicistat, Emtricitabine, Tenofovir alafenamide	Good clinical condition upon assessment
P7	M	50	1446	2007	Dolutegravir, Abacavir, Lamivudine	Good clinical condition upon assessment
P8	M	35	1196	2007	Emtricitabine, Tenofovir disoproxil fumarate, Efavirenz	Good clinical condition upon assessment
P9	M	36	820	na*	Dolutegravir, Abacavir,	Good clinical

					Lamivudine	condition upon assessment
P10	M	49	156	2015	Emtricitabine, Tenofovir disoproxil fumarate	Good clinical condition upon assessment

Key to Table 1. na*, unknown - the patient came to our unit as previously lost to follow up elsewhere and refused to give information of his previous medical history; viral load (copies/ml of serum) not available-the blood samples were freshly collected and have not been tested yet.

Peripheral blood samples (6-9 ml) from 10 HIV-1+ patients (Suppl. Table 1) were collected in heparinized tubes. PBMCs were isolated as described in the M&M of the manuscript. CD4+CD45RA+ cells were isolated from PBMCs using immunomagnetic beads as described (39). The isolated cells were cultured for 6h in the absence or presence of P/I, as described in the M&M of the manuscript.

At the end of the culture period, the cells where washed with PBS. An aliquot of cells was used for phenotyping using the mouse anti-human mAbs CD4-APC (clone RPA-T4), CD45RA-APC-H7 (clone 5H9) and CD45RO-PE-Cy7 (clone UCHL1) (BD Biosciences). Flow cytometric acquisition and analysis were performed on at least 10,000 acquired events per sample, using a Coulter EPICS-XL-MCL cytometer. The data were analyzed using the FlowJo V7.5 software (Tree Star Inc.).

The remaining cells were processed for quantitative real time PCR. The method and the primers used for ets-2 and β2-m are described in the M&M in the manuscript. The primers and conditions used for IL-2 expression are described (39).

To test whether the cells were infected (ref. S3), genomic DNA was extracted from 200 μl of peripheral blood of HIV-1 patients using a DNA extraction kit (MACHEREY-NAGEL). The genomic DNA yield and purity was determined by measuring absorbance at 260nm/280nm on a Quawell micro volume spectrophotometer Q3000 (Quawell Technology). A real-time PCR quantitative genomic DNA analysis was performed using 20 ng of genomic DNA from each sample on the Mx3000P Quantitative PCR System thermal cycler (Stratagene), using the SYBR-green fluorescence quantification technology (KAPA SYBR FAST qPCR Kit, Kapa Biosystems). The standard PCR conditions were 95^{0}C for 15 min followed by 40 cycles of 95^{0}C for 30 sec, 55^{0}C for 30 sec, and 72^{0}C for 30 sec. The results were analyzed using the MxPro software (Stratagene). The primers for HIV-1-tat gene were 5'-AGGAAGTCAGCCTAAAACTGC-3' and 5'-GCTCTTCGTCGCTGTCTCC-3', yielding a 125bp PCR product (ref. S3).

The results from the HIV-1-tat gene analysis showed that the cells were not infected (3) (data not shown).

The results (Supplementary Figure 3) show a similar pattern of Ets-2 expression in naive Th cells of the patients i.e. Ets-2 is expressed in unstimulated cells and its expression is reduced following mitogenic activation of the cells. Conversely, in the same cells, IL-2 is upregulated upon activation (39).

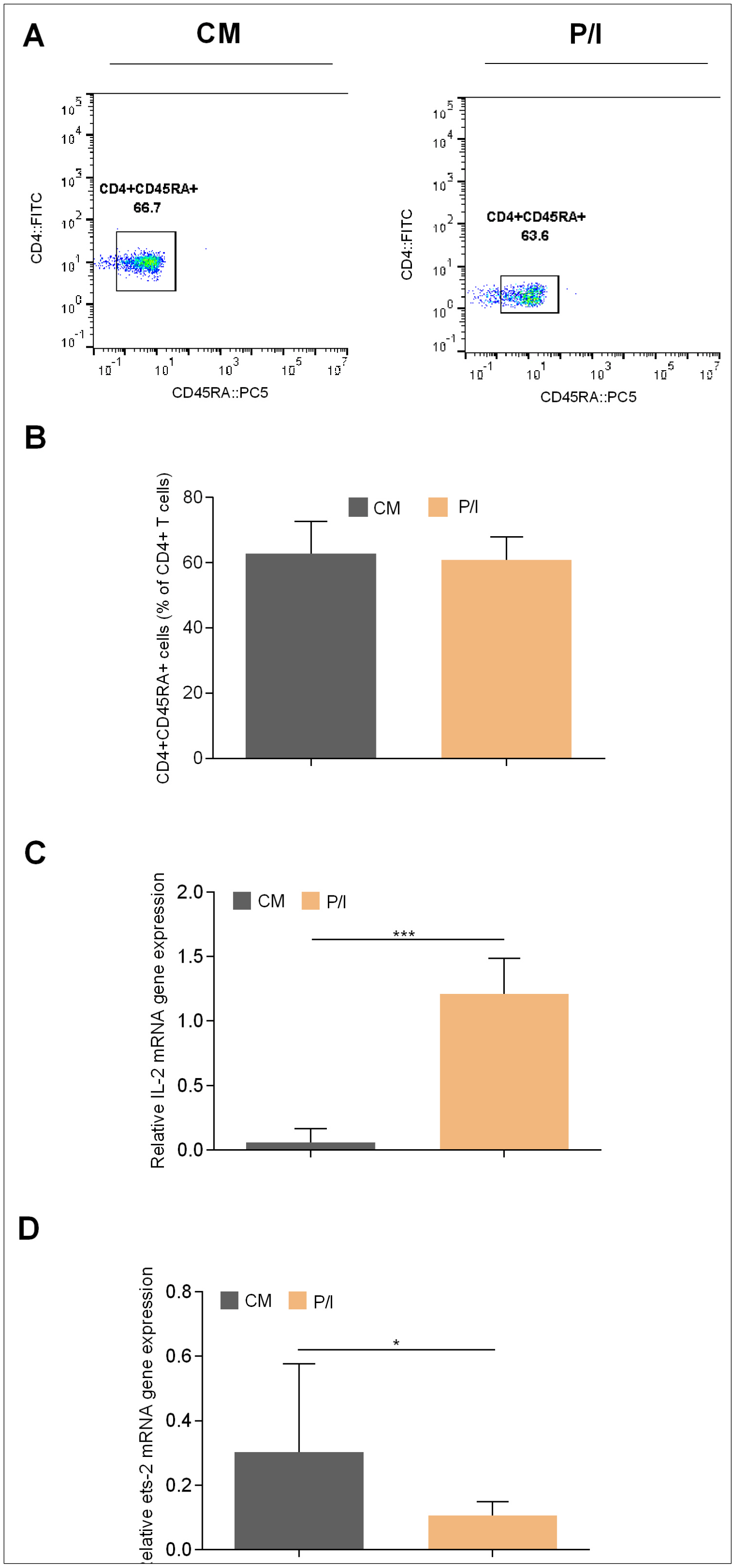

Supplementary Figure 3. Determination of Ets-2 expression in naive Th cells of HIV+ patients. (**A**) A representative flow cytometric analysis is shown for CD45RA+ naive CD4+ T-cells isolated from one patient before and after 6h stimulation with P/I. **(B)** The results [mean (SD)] of the analysis of CD45RA+ naive Th cells from 10 HIV-1+ patients. CM, culture medium; P/I, culture medium with P/I. IL-2 (**C**) and Ets-2 (**D**) mRNA levels measured by real time PCR in naive Th cells isolated from 10 HIV-1+ patients and cultured in CM or P/I for 6h. β2-m served as the normalizer gene for relative expression. The results are presented as the mean values (SD) from three independent experiments. Statistically significant differences are indicated by asterisks (*$p<0.05$, ***$p<0.001$, Student's t-test).

References used in Supplementary Material only

S1. EACS Guidelines, version 9, October 2017. http://www.eacsociety.org/files/guidelines_9.0-english.pdf

S2. INSIGHT START Study Group, Lundgren JD, Babiker AG, Gordin F, Emery S, Grund B, Sharma S, et al. Initiation of Antiretroviral Therapy in Early Asymptomatic HIV Infection. N Engl J Med (2015) **373**(9):795-807. doi: 10.1056/NEJMoa1506816.

S3. Blazkova J, Trejbalova K, Gondois-Rey F, Halfon P, Philibert P, Guiguen A et al. CpG methylation controls reactivation of HIV from latency. PLoS Pathog (2009) **5**(8):e1000554. doi: 10.1371/journal.ppat.1000554.
